# Assessing Inactivated Polio Vaccine Introduction and Utilization in Kano State, Nigeria, April–November 2015

**DOI:** 10.1093/infdis/jix044

**Published:** 2017-07-01

**Authors:** Lynda U. Osadebe, Adam MacNeil, Hashim Elmousaad, Lora Davis, Jibrin M. Idris, Suleiman A. Haladu, Olorunsogo B. Adeoye, Patrick Nguku, Uneratu Aliu-Mamudu, Elizabeth Hassan, John Vertefeuille, Peter Bloland

**Affiliations:** 1 Global Immunization Division, Centers for Disease Control and Prevention, Atlanta Georgia;; 2 Nigerian Stop the Transmission of Polio Program, AbujaNigeria;; 3 Nigeria Field Epidemiology and Laboratory Training Programs; and; 4 National Primary Health Care Development Agency

**Keywords:** Inactivated polio vaccine, vaccination introduction, immunization information systems, routine immunization.

## Abstract

**Background.:**

Kano State, Nigeria, introduced inactivated polio vaccine (IPV) into its routine immunization (RI) schedule in March 2015 and was the pilot site for an RI data module for the National Health Management Information System (NHMIS). We determined factors impacting IPV introduction and the value of the RI module on monitoring new vaccine introduction.

**Methods.:**

Two assessment approaches were used: (1) analysis of IPV vaccinations reported in NHMIS, and (2) survey of 20 local government areas (LGAs) and 60 associated health facilities (HF).

**Results.:**

By April 2015, 66% of LGAs had at least 20% of HFs administering IPV, by June all LGAs had HFs administering IPV and by July, 91% of the HFs in Kano reported administering IPV. Among surveyed staff, most rated training and implementation as successful. Among HFs, 97% had updated RI reporting tools, although only 50% had updated microplans. Challenges among HFs included: IPV shortages (20%), hesitancy to administer 2 injectable vaccines (28%), lack of knowledge on multi-dose vial policy (30%) and age of IPV administration (8%).

**Conclusion.:**

The introduction of IPV was largely successful in Kano and the RI module was effective in monitoring progress, although certain gaps were noted, which should be used to inform plans for future vaccine introductions.

As part of the polio eradication and endgame strategy, inclusion of at least 1 dose of inactivated polio vaccine (IPV) into countries routine immunization (RI) schedules is recommended [1, 2]. Additionally, Nigeria developed a polio endgame strategy that focuses on strengthening RI services and collecting high-quality immunization data [3]. This is in line with 2 of the guiding principles of polio legacy planning, which are applying the successes of polio eradication to RI and enabling countries to use polio resources to strengthen existing public health functions [4].

To assist with monitoring and improving performance, Nigeria developed an RI-specific data module that was integrated into the National Health Management Information System (NHMIS). The NHMIS is operated on an electronic open-source software called the District Health Information System version 2 (DHIS2) [5]. The RI module is a computer-based platform that allows Local Government Areas (LGAs), states, and federal government health agencies to visualize key indicators using a dashboard and enable access to RI service delivery data within 24 hours of data entry. The RI module was introduced in Kano State in November 2014 as a pilot to evaluate its feasibility and identify problems before implementing the module nationwide. Kano State introduced IPV in March 2015, presenting an opportunity to assess the utility of the RI module to investigate factors that contribute to or inhibit effective vaccine introduction at LGAs and health facilities (HFs). Two approaches were used to assess IPV introduction: (1) analysis of IPV coverage data collected through the NHMIS, and (2) a questionnaire administered in 60 HFs in 20 LGAs to identify factors that facilitated or hindered IPV introduction.

## METHODS

### Data Source

Data from the NHMIS were downloaded for two time periods: (1) August 2015 for the months of March to June 2015 and (2) February 2016 for the months of July to November 2015. The first time point (August 2015) was to enable site selection based on IPV introduction using April 2015 as the target month; data extraction occurred before the field assessment in January 2016. April was used as the target month to adjust for time lag between IPV statewide launching and actual introduction at the service delivery level. The latter time period, February 2016, which occurred after the field assessment, was used to assess IPV utilization. Data on the total number of children vaccinated with IPV and a third dose of pentavalent vaccine containing diphtheria–tetanus–pertussis–*Haemophilus influenzae* type b–hepatitis B antigens (Penta3) from March to November 2015 were retrieved to assess IPV utilization. Because Penta3 and IPV are given to a child at the same visit, the concordance between antigens was used to determine IPV uptake and utilization.

### Selection of Local Government Areas and Health Facilities

**Figure 1.**
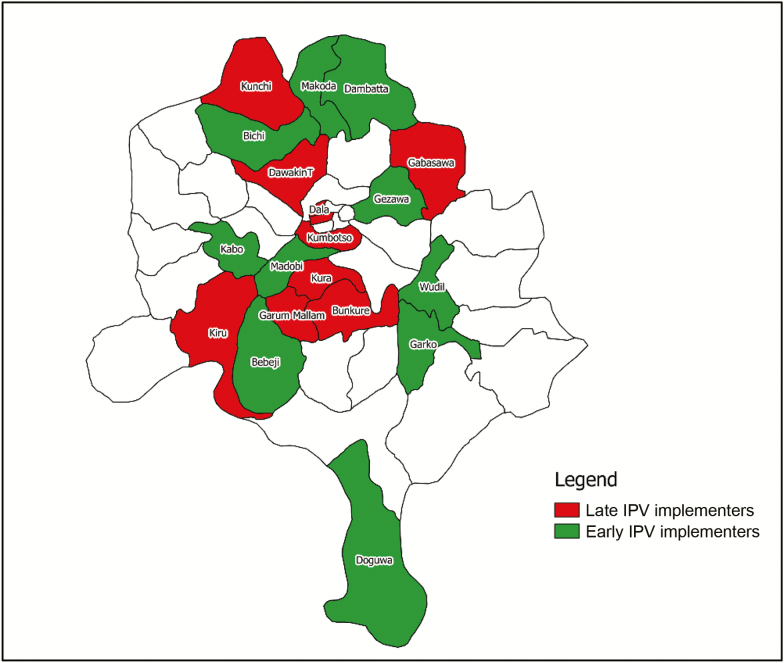
Map showing Kano State local government areas sampled in this assessment. Abbreviation: IPV, inactivated polio vaccine.

Three hundred sixty-one of 1156 (31.2%) HFs reported administering IPV in April 2015. To determine factors related to early vaccine adoption, 10 LGAs were randomly selected from LGAs that had introduced IPV before or during April 2015, and 10 were randomly selected from LGAs that introduced IPV after April 2015 (Figure 1). Five interview sites within each LGA were selected. The sites included the LGA health office, the cold store (if separate from the LGA office), and 3 HFs. The 3 HFs were selected based on doses of IPV, third doses of oral poliovirus vaccine (OPV3), and doses of Penta3 administered. For the 10 LGAs that had introduced IPV by April 2015, 30 HFs were randomly selected from a total of 213 HFs, and the selection criteria were based on (1) HFs administering IPV, OPV3, and Penta3 to equal number of children; (2) HFs administering OPV3 and Penta3 to a higher number of children than IPV; or (3) HFs administering OPV3 and Penta3 but not IPV to children. Ten HFs were randomly selected from each aforementioned category. Thirty HFs were also randomly selected from a total of 279 HFs from the 10 LGAs implementing after April. These HFs were selected based on (1) HFs administering equal amounts of OPV3 and Penta3 or (2) HFs administering unequal doses of OPV3 and Penta3.

### Data Field Collection

Data collection occurred January 11–15, 2016, and was preceded by a 2-day training of 25 field staff. Data were collected using questionnaires and a record review of Penta1, Penta3, and IPV administered at LGAs and HFs. For the purpose of this assessment, the staffs were interviewed as teams with the LGA immunization officer as the main LGA respondent, and the RI focal person was the main respondent for the HF questionnaires.

Two standardized questionnaires modeled after the Kano State Data Quality Supportive supervision and World Health Organization new vaccine Post Introduction Evaluation (PIE) tools [6] were used to interview LGA and HF staff. Questions were grouped into 5 categories: (1) human resources, (2) IPV introduction planning, (3) IPV training, (4) vaccine logistics, and (5) healthcare workers’ knowledge. Both questionnaires were piloted in an LGA office and 2 HFs in Abuja, the Nigerian capital. Data were collected both by paper and electronically using Open Data Kit [7]. Project staff reviewed data daily for any errors in data collection onsite and conducted spot-checks before downloading data.

### Ethical Review

This assessment was classified as a routine public health program evaluation by the Kano State Medical Review Board and by the US Centers for Disease Control and Prevention.

## RESULTS

**Figure 2.**
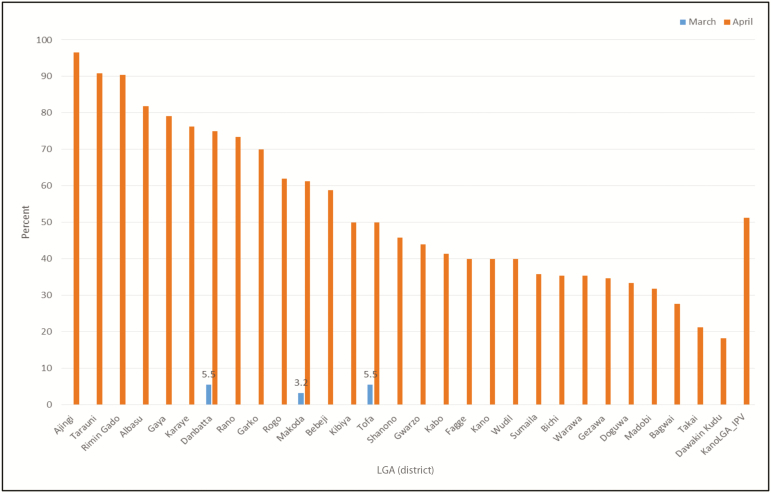
Proportion of health facilities administering inactivated polio vaccine by local government area (LGA), March–April 2015.

**Figure 3.**
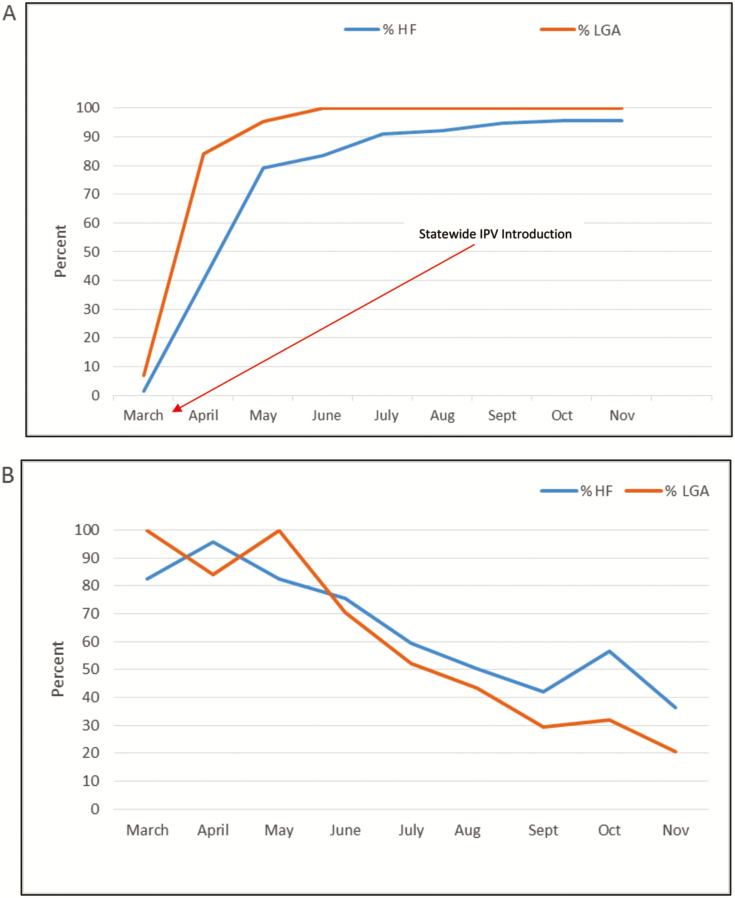
*A,* Proportion of health facilities (HFs) and local government areas (LGAs) implementing inactivated polio vaccine (IPV) in Kano State, March–November 2015. *B,* Proportion of health facilities and LGAs with >10% discrepancies in administered doses of IPV and pentavalent vaccine containing diphtheria–tetanus–pertussis– *Haemophilus influenzae* type b–hepatitis B antigens (Penta3) in Kano State, March–November 2015.

Kano State introduced IPV in March 2015; as of April 2015, 29 (66%) of 44 LGAs had ≥20% of HFs administering IPV (Figure 2). By June 2015, all 44 LGAs were administering IPV (Figure 3A). The discordance between the number of children given IPV and the number given Penta 3 demonstrated a downward trend over the same time period at the LGA and the HF level (Figure 3B).

### Local Government Area Findings From the Survey

#### Staff Characteristics

Out of 71 staff working at the 20 LGA offices assessed, 50 were interviewed. Respondents were interviewed as an LGA team of 2–5 people. Forty-four (88%) of the Respondents were cold chain officers (n = 19), LGA (district) immunization officers (n = 15), and monitoring & evaluation officers (n = 10). Forty-six of the respondents attended IPV training, with 29 attending before April, and all 20 LGA offices had at least 1 staff member who attended the IPV training. Staff interviewed had worked an average of 4.4 years in their position (range = 6 months to 13 years).

### Factors That Affect Inactivated Polio Vaccine Introduction

#### Inactivated Polio Vaccine Introduction, Related Timelines, and Training

As shown in Table 1, the majority of LGAs had introduced IPV by April 2015 and had received all updated RI tools (the NHMIS supplementary 2014 forms, tally sheets, child register, and immunization cards showing IPV) before introduction. Eleven (55%) LGAs had IPV training within a month of introduction; the average time between training and introduction was 15 days before introduction, with the maximum time being 2 months before or after introduction.

**Table 1.** Characteristics of the 20 Kano State Local Government Areas Sampled in This Assessment, January 2016

LGA characteristics (N = 20)	No. (%)
IPV introduced by April	16 (80)
Received updated RI tools before introduction	14 (70)
Received updated RI tools by January 2016 (day of assessment)	19 (95)
IPV integrated with other programs	10 (50)
Availability of an introduction plan	
LGA specific	8 (40)
National/regional	7 (35)
IPV training	
>1 mo before introduction	1 (5)
Within 1 mo of introduction (ideal)	11 (55)
>1 mo after introduction	8 (40)
State training included:	
Public HF workers	16 (80)
Private HF workers	3 (5)
State funded IPV training	16 (80)
Rating of IPV training, mean (range)	86.5 (70–100)
Staff knowledge	
Age of IPV administration	15 (75)
Correct wastage rate formula	14 (70)
Correct vaccine coverage formula	18 (90)
IPV multi-dose vial policy	
Discard after 28 d	6 (30)
Discard after 6 hrs/RI session	10 (50)
Cold chain management	
Acquired new freezer	6 (30)
Repaired existing freezer/fridge	3 (15)
Acquired a power generator/solar system	3 (15)
Stored IPV in state cold store	1 (5)
Other	11 (55)
Faulty equipment (after IPV introduction)	
Faulty fridge/freezer	3 (15)
Vaccine logistics:	
*Vaccine requirement determination*	
Predetermined by state	3(15)
Weekly checks	4 (20)
Monthly coverage rate	6 (30)
Quarterly forecast	3 (15)
Target population	5 (25)
*IPV vaccine supply*	
Received IPV by April	17 (85)
Notified health facilities by April	16 (80)
*Stockout*	1 (5)
Advocacy	
LGA launch	6 (30)
Community engagement	18 (90)
Staff general impression	
IPV introduction improved EPI	15 (75)
Smooth introduction no problems	18 (90)
Overall rating of introduction, mean (range)	87.7 (65–100)
Lessons learned	
Expand content of health workers training	6 (30)
Early community engagement	9 (45)

Abbreviations: HF, health facility; IPV, inactivated polio vaccine; LGA, local government area; RI, routine immunization; EPI, expanded program on immunization.

### Local Government Area Workers’ Knowledge

Staff from 15 (75%) of the LGAs knew the correct age of IPV administration (14 weeks), and staff from 16 (80%) of the LGAs knew the existing IPV multidose policies. Staff from 18 (90%) of the LGAs knew the vaccine coverage formula.

### Inactivated Polio Vaccine Logistics and Utilization

Seventeen (85%) of the LGAs had received their first IPV supply by April 2015. Kano State vaccine store supplied IPV directly to facilities, bypassing LGA vaccine storage sites, because only 5 (25%) LGAs sampled reported distributing vaccines to HFs. Only 1 LGA reported IPV stock-out for a week because of no supply at the state.

Two LGAs (Kumbotso and Makoda) illustrate some of the problems with actual administration of IPV. Only 5 of 24 (16.1%) HFs in the Kumbotso LGA offered IPV by April 2015 because of the state did not provide vaccine. In the Makoda LGA, 15 HFs (40%) did not offer IPV in RI sessions by April because healthcare workers viewed it unnecessary due to a polio campaign held in October 2014.

### Penta3 and Inactivated Polio Vaccine Coverage

From the data extracted at LGA offices, 17 LGAs had complete data for both IPV and Penta3 coverage in September–November 2015. Although IPV and Penta3 should be administered at the same time, the correlation between the 2 antigens’ coverage was variable(correlation coefficients = 0.45 in September, 0.79 in October, and 0.52 in November). Five (29.4%) LGAs had >10% discrepancy in IPV and Penta3 coverage, with values as high as 41%.

### Advocacy and Community Engagement

All LGA staff reported IPV vaccine being well accepted by the community and healthcare workers, as evidenced by reported increases in client turnout due to children >14 weeks receiving IPV following introduction. Eighteen LGAs conducted community outreach activities (radio announcements and information sessions).

### General Impression of Inactivated Polio Vaccine Introduction

Respondents were asked to rate their impression of the success of the IPV introduction using a scale of 0 (worst impression) to 100 (best impression); respondents gave an average rating of 87.7 (range = 65–100). From the staff perspective, lessons learned primarily grouped into 2 broad categories: (1) community education, including timing between engagement and IPV introduction, addressing concerns about multiple injections, and making information, education, and communication materials available in local dialects; and (2) health workers training.

### Health Facility Results From the Field Assessment

Three HFs were sampled from each of the 20 LGAs, for a total of 60 HFs. Sixty-three staff were interviewed out of a total of 174 with RI duties. Sixty (95%) of the staff interviewed were either the clinic in-charge (34; 57%) or the RI focal person (26; 43%). The clinic in-charge was responsible for all services rendered in the facility, whereas the RI focal person was responsible for immunization services. The average number of staff with RI-specific duties working per facility was 3, with a range of 1–10.

As shown in Table 2, 23 (38%) HFs had implemented IPV by April 2015, making them “timely” HFs, whereas the remaining 37 (62%) HFs implemented after April, making them “delayed” HFs. Also the delayed health facilities reported more IPV shortages, parental refusal, and increased workload than their timely counterparts, although the difference did not reach statistical significance. Given that there were no further differences between HF categories, subsequent results will be reported by looking at all 60 HFs regardless of aforementioned categories, and with applicable differences highlighted.

**Table 2.** Characteristics of the 60 Health Facilities Sampled in This Assessment, January 2016

HF characteristics (N = 60)	Timely HF^a^ (n = 23) no. (%)	Delayed HF^b^ (n = 37) no. (%)
Facility type		
Rural health center	10 (43.5)	13 (35.1)
Health post	5 (21.7)	10 (27.0)
Government hospitals	7 (30.4)	12 (32.4)
Dispensary	1 (4.3)	2 (5.4)
Use of updated tools		
Child immunization card	23 (100)	37 (100)
Tally sheet	23 (100)	35 (94.5)
Child immunization register	18 (78.3)	32 (86.5)
Availability of key RI forms and documents		
National immunization schedule	17 (73.9)	35 (94.6)
IPV vaccine guideline	14 (60.9)	17 (46.0)
HF monthly summary form	21 (91.3)	36 (97.3)
NHMIS supplementary form (2014)	22 (95.7)	37 (100)
Vaccine utilization form (VM1a)	21 (91.3)	37 (100)
Vaccine stock ledger	22 (95.7)	34 (91.9)
Updated microplan seen	11 (47.8)	21 (56.8)
Supervisory book	23 (100)	37 (100)
IPV training (58)^c^		
Vaccine samples shown	20 (90.9)	32 (88.9)
Administration skills practiced	20 (90.9)	33 (91.7)
IPV guidelines given (47)^c^	18 (90)	29 (80.6)
FAQs given (18)^c^	6 (40)	12 (42.9)
Education materials given	9 (39.1)	17 (46)
Outreach materials given	14 (60.9)	17 (46)
Training rating, mean (range)	84.5 (60–100)	87.8 (60–100)
Outreach session changes after IPV introduction (59)^c^		
No changes	20 (87)	26 (72.2)
More vaccine carriers	1 (4.3)	1 (2.8)
Increase in session time	0	1 (2.8)
More personnel	0	1 (2.8)
More community engagement	1 (4.3	4 (11.1)
IPV vaccine distribution (59)^c^		
States supplies (Push system)	12 (54.5)	23 (62.2)
LGA supplies	8 (36.4)	11 (29.7)
HF staff collects from LGA/ state cold room	3 (13.6)	3 (8.1)
IPV shortage since introduction (12)^c^	**2 (8.7**)	**10 (27.0**)
Staff knowledge (59)^c^		
Know IPV multi-dose vial policy	13 (56.5)	21 (58.3)
Don’t know	5 (21.7)	13 (36.1)
IPV administration (60)^c^		
Correct age	20 (86.9)	35 (94.6)
Correct route	22 (95.7)	37 (100)
IPV coadministration (59)^c^		
With Penta3 or OPV3 only	12 (52.1)	13 (35.1)
With both Penta3 and OPV3	11(47.8)	23 (63.9)
Parent refusal	**0**	**7 (18.9**)
Effect of IPV introduction		
Increased staff work load	**2 (8.7**)	**16 (43.2**)

The bolded figures indicate a notable difference between both groups; the “Delayed” health facilities reported more IPV shortages, parental refusal, and increased workload than their “Timely” counterparts, although difference did not reach statistical significance.

Abbreviations: HF, health facility; IPV, inactivated polio vaccine; LGA, local government area; NHMIS, National Health Management Information System; OPV3, third dose of oral polio vaccine; Penta3, pentavalent vaccine containing diphtheria–tetanus–pertussis–*Haemophilus influenzae* type b–hepatitis B antigens; RI, routine immunization.

^a^“Timely” introduction is defined as health facilities that implemented IPV on or before April 2015 and vaccinated an equal number of children with IPV and Penta3 at the time of implementation.

^b^“Delayed” introduction refers to health facilities that implemented IPV after April and where the discrepancy between Penta3 and IPV doses administered was ≥10%. The delayed category also includes 10 health facilities that did not implement IPV in April 2015.

^c^Number in parentheses are the health facilities whose staffs responded to a question.

### Availability of Updated Immunization Paper Recording Tools and Forms

All 60 HFs were using updated childhood immunization cards by April 2015. All of the timely HFs were using updated tally sheets compared with 35 (94.5%) of their delayed counterparts. As for the child immunization register, 10 HFs in 7 LGAs had no updated child immunization register capturing the IPV column on the day of the assessment (January 2016). Notably, all 3 HFs assessed in 1 LGA had no updated immunization register. One HF was not using any of the updated tools, despite availability of tools. Only 32 (53.3%) HFs had an updated microplan during the interview; 22 (68.8%) of these were either health posts or rural health centers.

### Inactivated Polio Vaccine Training

At least 1 staff from 59 of 60 of the HFs attended the training on IPV administration; the remaining HF was a small health post serving approximately 5 children per week. All staff who attended training were comfortable administering the vaccine after training.

### Vaccination Service Delivery

Of the 60 HFs, 56 (93%) HFs integrated other services with immunizations such as health education and patient care. All 60 HFs offered IPV in both fixed and outreach sessions held in December 2015, a month before the assessment month. Thirteen (21.7%) HFs made changes to outreach sessions because of IPV introduction, including increased session time, community engagement to educate caregivers about IPV, and requests for more staff.

### Correlation Between Penta3 and Inactivated Polio Vaccine Doses Extracted From the Health Facility Summary Form

**Figure 4.**
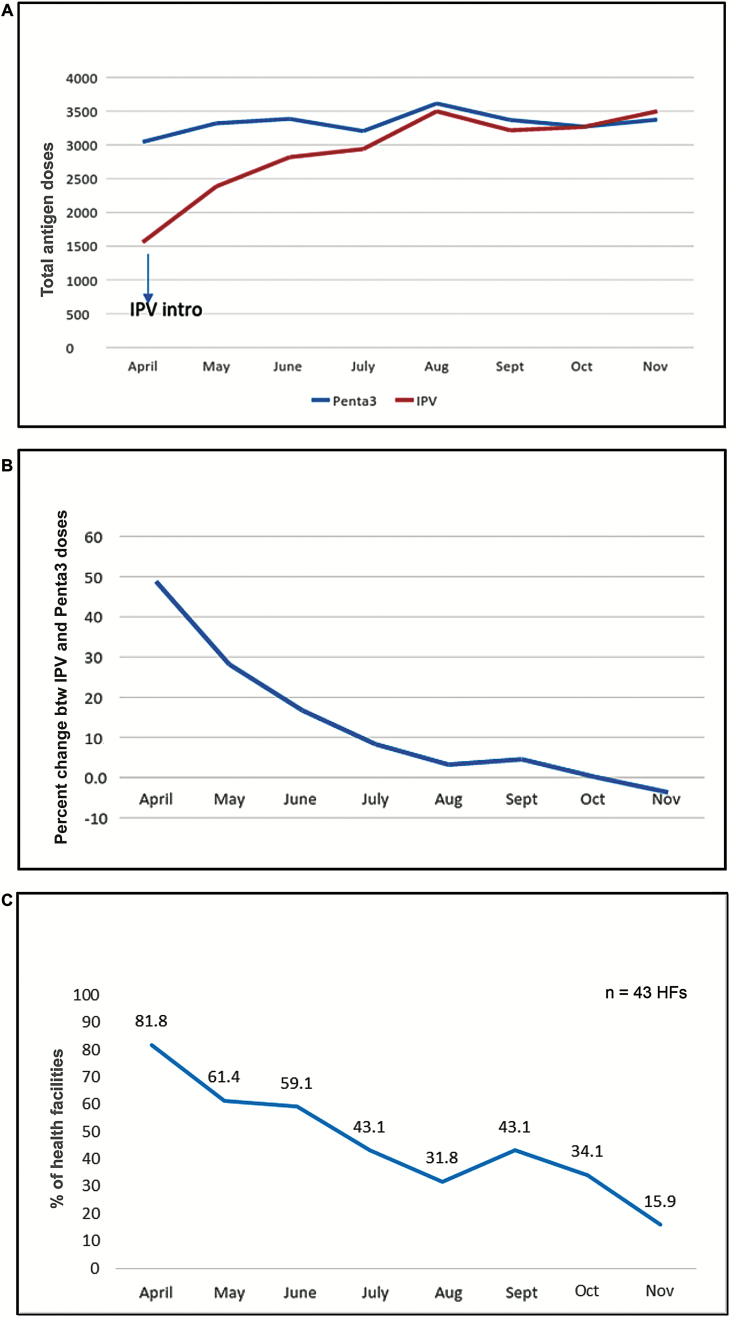
*A,* Total doses of inactivated polio vaccine (IPV) and pentavalent vaccine containing diphtheria–tetanus–pertussis–*Haemophilus influenzae* type b–hepatitis B antigens (Penta3) administered in the 60 health facilities assessed. *B,* Percentage change between IPV and Penta3 doses administered, April–November 2015. *C,* Proportion of health facilities (HFs) with >10% discordance between IPV and Penta3 doses, April–November 2015.

Figure 4A shows the decreasing trend in discordance between IPV and Penta3 doses administered with time. The initial discrepancy can be attributed to the months of April to June 2015 (the first quarter of IPV introduction) (Figure 4B). The downward trend in discrepancy between both antigens can be further explained by a decrease in the number of HFs with >10% discrepancy from April to November (Figure 4C). In April, 35 (81.8%) HFs had >10% discordance compared with 7 (15.9%) in November 2015.

### Vaccine Logistics

Only 6 (10%) HFs assessed reported IPV wastage rate for April–November 2015; the rest reported not computing the indicator. Thirteen (21.7%) HFs had no source of power supply; thus vaccines were stored in cold boxes. Ten of these were either health posts or rural health clinics; and 3 were general hospitals serving a range of 21–40 children/week. Four HFs reported having faulty equipment; 2 of these were large hospitals. Thirty-five (58%) HFs reported receiving vaccines directly from the state, and staff from 6 HFs collected vaccines from the state/LGA cold room either weekly or biweekly (5 of these were either health posts, rural health centers, or dispensaries). Twelve (20%) of the HFs reported IPV shortages. Three of these reported durations ranging from 1 week to 2 months and cited reasons such as lack of state supply, lack of transport, and increased migration from surrounding communities.

### Staff Knowledge and Caregiver Refusal

Staff from 18 (30.5%) HFs did not know the IPV multidose open-vial policy. Staff from 5 (8.3%) HFs did not know the age of IPV administration; answers ranged from 3 weeks to 18 weeks. Staff from 34 (57.6%) of the HFs correctly stated that IPV can be administered with Penta3 and OPV3; however, staff from 8 HFs will only give 2 injectable vaccines during the same session because of pain to child and increased risk for side effects. Staff from 17 (28.3%) HFs reported they were unwilling to give >2 injectable vaccines at a single visit. Seven (11.6%) HFs reported episodes of caregiver refusal; reasons cited were resistance to administration of multiple injectable vaccines at a single visit (n = 2), resistance to administration of multiple polio vaccines (n = 2), distrust of new vaccines (n = 2), and concern about pain (n = 1). These 7 HFs were all delayed HFs. Thirty-five (58.3%) HFs had some form of educational material such as posters, and 31 of these received the materials by April 2015. Staff from 30 (50%) of the HFs participated in community engagement activities such as advocacy and training of community volunteers.

### Impression of Inactivated Polio Vaccine Introduction

Respondents at 58 (96.6%) HFs thought IPV introduction went smoothly, although those from 18 (30%) of the HFs, 16 of which were delayed HFs, stated it resulted in an increase in staff workload.

### Utility of National Health Management Information Systems Reporting Platform

To determine the dependability of the NHMIS reporting platform, IPV coverage and number of children given IPV were used for comparison between data reported on the platform and that obtained from the summary forms at the LGA offices and HFs sampled. Ten of the 20 LGAs sampled had >10% discrepancy in IPV coverage between both data sources; 2 of these LGAs (Bebeji and Kumbotso) had consistent discordance the entire quarter (September–November 2015) (Table 3).

**Table 3.** Absolute Difference in Inactivated Polio Vaccine Coverage Reported on National Health Management Information System and Recorded on the Local Government Area Summary Forms in 20 Kano State Local Government Areas, September–November 2015

LGA	September			October			November		
	NHMIS	Survey	Difference	NHMIS	Survey	Difference	NHMIS	Survey	Difference
**Bebeji**	77.9	NR	**77.9**	80.5	NR	**80.5**	78.7	NR	**78.7**
Bichi	90.9	102.0	**−11.1**	79.5	66.0	**13.5**	99.8	91.0	8.8
Bunkure	72.9	101.4	**−28.5**	69.2	86.2	**−17.0**	89.4	92.9	−3.5
Dala	105.6	110.0	−4.4	119	126.0	−7.0	116.4	125.0	−8.6
Dambatta	94	93.2	0.8	80.8	99.1	**−18.3**	94.5	NR	**94.5**
DawakinTofa	85.8	98.0	**−12.2**	116.3	117.0	−0.7	107.7	101.0	6.7
Doguwa	133.5	135.0	−1.5	134.8	132.0	2.8	134.1	136.0	−1.9
Gabasawa	113.2	119.0	−5.8	119.6	120.0	−0.4	88.8	90.0	−1.2
Garko	88.2	89.4	−1.2	95.7	93.9	1.8	98.4	99.8	−1.4
GarunMallam	94.8	94.0	0.8	90.6	88.0	2.6	91.7	90.0	1.7
Gezawa	122.7	115.0	7.7	121.9	107.0	**14.9**	91.9	98.0	−6.1
Gwale	87	92.0	−5.0	101.6	101.6	0	100.2	98.3	1.9
Kabo	120.3	120.0	0.3	123.3	124.0	−0.7	66.5	67.0	−0.5
Kiru	98.9	107.0	−8.1	113.4	112.0	1.4	91.7	100.0	−8.3
**Kumbotso**	79.5	NR	**79.5**	101.9	NR	**101.9**	108.1	NR	**108.1**
Kunchi	136	86.0	**50.0**	93.8	94.0	−0.2	99.7	95.0	4.7
Kura	98.5	78.8	**19.7**	104.7	81.5	**23.2**	99	93.1	5.9
Madobi	96.7	112.0	**−15.3**	91.5	96.0	−4.5	95.9	96.0	−0.1
Makoda	85.9	83.0	2.9	88.2	81.8	6.4	83.8	82.9	0.9
Wudil	90.3	85.9	4.4	82.9	81.0	1.9	88.1	87.0	1.1

Bold fonts show discrepancy >10% or below −10%. Discrepancy was calculated as the absolute difference between the IPV coverage computed on the NHMIS platform and that retrieved from the local government area (LGA) summary form at the 20 LGA offices during the assessment. Districts bolded are the ones with consistent discordance between both sources over a quarter (September–November 2015).

Abbreviatons: LGA, local government area; NHMIS, National Health Measurement Information System; NR, not recorded.

**Figure 5.**
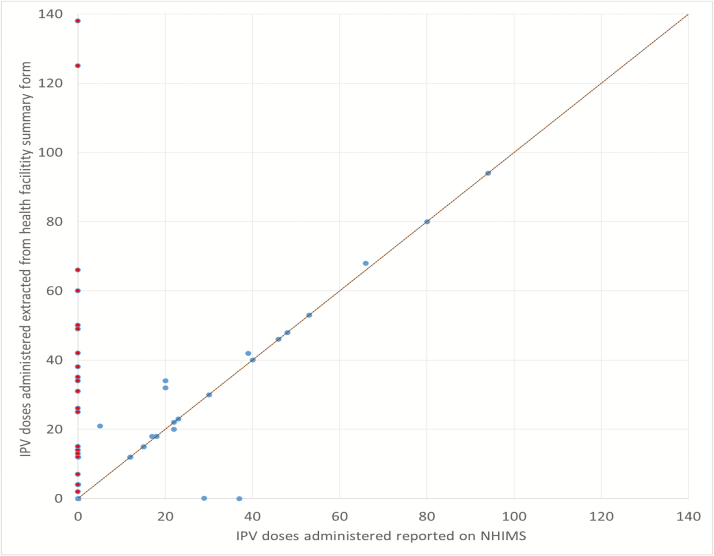
Correlation between the number of children vaccinated with inactivated polio vaccine (IPV) recorded on the health facility summary form and those reported in the National Health Measurement Information Systems (NHMIS) platform in April 2015.

The correlation between the number of children vaccinated with IPV recorded on the HF summary form and that reported in the NHMIS in April 2015 was weak, with a correlation coefficient of 0.40. This may be explained by the 22 HFs whose data were not captured in the NHMIS when site selection was conducted (Figure 5).

## DISCUSSION

The introduction of IPV was successful in the majority of LGAs based on preimplementation activities (availability of updated tools, timing of staff training, and acceptance by both the community and health workers). Similarly, by July 2015 there was strong concordance between Penta3 and IPV doses administered.

Intensive community engagement assisted in the successful introduction. These activities included hosting advocacy meetings, training community volunteers, and using media outlets. A few of the staff suggested providing outreach materials in local dialects and improving the timing of community engagements, which appeared to intensify in April after the official launch in March 2015.

Forty percent of LGAs developed a specific LGA introduction plan, in agreement with the World Health Organization’s recommendations for introducing new vaccines [6]. Development of an introduction plan provides an opportunity to revise microplans, develop strategies for subpopulations, and identify and address gaps in the existing RI system. Such gaps will give insights on areas to include in the new vaccine training for healthcare workers.

This assessment highlights gaps in healthcare worker knowledge about the IPV multidose vial policy and the coadministration of IPV with other vaccines, which may explain partially the reluctance of health workers to give multiple injectable vaccines at a single visit. A literature review by Wallace et al [8], which included 44 articles from 39 predominantly upper- income countries that looked at provider attitude and multiple injections for infants results, revealed many health workers expressed reluctance to increase the number of injectable vaccines they administered in a single visit when a new vaccine was introduced. Similarly, an assessment of the IPV introduction in The Gambia found that 9.9% of health workers and 35.7% of infant caregivers expressed concerns about children receiving >2 injectable vaccines in a single visit [9].

For Kano State, resistance to administering multiple injectable vaccines at a single visit may have implications for the recent introduction of pneumococcal conjugate vaccine (PCV), an injectable vaccine given at 14 weeks along with Penta3 and IPV. Strategies to tackle healthcare workers’ and caregivers’ concerns about administering multiple injectable vaccines at a single visit must be developed to ensure the success of IPV and PCV adoption, including assuring healthcare workers about the safety of new vaccines, educating them about the benefits of giving several vaccinations in 1 visit, and strengthening the surveillance for adverse events following immunization [2]. These strategies were used in South Africa and Brazil during IPV introduction and were valuable in engaging healthcare workers [10, 11].

Because of polio endgame strategy, there has been an emphasis on microplanning and reaching every ward, but our assessment indicates gaps in the implementation of this strategy in Kano State. Only half of HFs in this assessment had an updated microplan. Interestingly, most of these HFs have had several supportive supervision visits, indicating the potential need for refresher supervisory training in microplans and potentially broader issues with the current knowledge of supervisors.

In a few LGAs, there were reports of IPV shortage; the main reason noted was delayed supply from the state. In addition, the mechanism of the existing vaccine distribution system (the Push system) is poorly understood by the LGA and HF staff interviewed. Ideally, the Push system should relieve LGAs and HFs from handling vaccine transportation issues, thereby allowing LGA staff to intensify other activities such as data quality assurance, supportive supervision, and community engagement. For now, it appears Kano State has yet to maximize the utility of such a system.

This assessment demonstrates the utility of the NHMIS reporting system to inform programmatic decisions at the service delivery levels. The trends in IPV uptake and Penta3 and IPV discordance were similar based on paper review of HF data. However, comparison of NHMIS data to that obtained from HFs underscores the issue of late and incomplete reporting. Using April as the target month for IPV implementation, the NHMIS platform misclassified 6 LGAs and 22 HFs as late IPV implementers. Notably the data for site selection were retrieved in August 2015, 5 months after IPV statewide introduction. Data entry error at the LGA may also be a reason for the discrepancies between both sources. Kano State has intensified strategies to improve both reporting timeliness and data quality. Strategies include performing data quality supportive supervision both at LGAs and HFs and investigating reasons for late submission by LGAs or HFs. These investigations are usually initiated by the LGA immunization officer and the monitoring-and-evaluation officer of the implicated LGA. It will be worthwhile to monitor the impact of such strategies in improving RI delivery and data quality.

In 2012, Nigeria embarked on activities to strengthen RI, which included training health workers, updating reaching- every-ward microplans, intensifying supportive supervision, ensuring vaccine availability, using data for action, and leveraging supplementary immunization activities [12]. With the recent cases of polio discovered in the country, this may be the ideal time to ensure these strategies are effectively implemented at the service delivery levels and monitoring and evaluation plans are instituted to appraise the impacts of these strategies. Based on this assessment, the NHMIS can play a useful role in these future efforts to strengthen Nigeria’s RI performance.

## Notes

***Acknowledgments.*** The authors thank Dr Sharif Yahaya Musa, the Kano State immunization officer; Eric Wiesen, CDC Nigeria Polio Team lead; Dr Chime Nnadi, CDC Nigerian Polio Team; Sara Andrist, CDC Strategic Information Team; and Drs Endie Waziri and Gidado, NSTOP, for their guidance and support of this work. We also thank Dr Joseph Lubwama for the initial data analysis and the NSTOP staff and the NFLTEP fellows who participated as field data collectors and supervisors. Finally, the authors also acknowledge all the LGA and HF staff that participated in this assessment.

***Financial support.*** This work was supported by funds from the Centers for Disease Control and Prevention.

***Supplement sponsorship.*** This work is part of a supplement coordinated by the Task Force for Global Health with funding provided by The Bill and Melinda Gates Foundation and the Centers for Disease Control and Prevention.

***Potential conflicts of interest***.All authors: No reported conflicts. All authors have submitted the ICMJE Form for Disclosure of Potential Conflicts of Interest. Conflicts that the editors consider relevant to the content of the manuscript have been disclosed.
